# Does Ovariectomy Affect the Mechanics of the Mandibular Alveolar Bone Structure of Wistar Rats Subjected to Tooth Loss and Modified Diet?—A FEA Study

**DOI:** 10.3390/biology13110906

**Published:** 2024-11-06

**Authors:** Camila C. Furlan, Alexandre R. Freire, Beatriz C. Ferreira-Pileggi, Luciane N. O. Watanabe, Paulo R. Botacin, Felippe B. Prado, Ana Cláudia Rossi

**Affiliations:** 1 Biosciences Department, Piracicaba Dental School, University of Campinas (UNICAMP), Piracicaba 13414903, SP, Brazil; camilacarrillofurlan@gmail.com (C.C.F.); alerfreire@gmail.com (A.R.F.); biacarmonaf@gmail.com (B.C.F.-P.); lucianewatanabe@gmail.com (L.N.O.W.); fbprado@unicamp.br (F.B.P.); 2 Department of Basic Sciences, Araçatuba Dental School, São Paulo State University Júlio de Mesquita Filho—UNESP, Aracatuba 16015050, SP, Brazil; paulo.botacin@unesp.br

**Keywords:** osteoporosis, mandible, finite element method, diet, tooth

## Abstract

**Simple Summary:**

The aim of this study was to evaluate the mechanical effect of ovariectomy, diet (hard or soft food) and tooth extraction on the bone structure of the mandible of Wistar rats. The association of the mechanical test and simulation by finite element method showed that the diet and dental condition interfered in the distribution of stresses on the rat mandible. The presence of teeth shows a protective mechanical effect on the mandible.

**Abstract:**

The aim of this study was to evaluate the mechanical effect of ovariectomy, diet, and tooth extraction on the bone structure of the mandible of Wistar rats. Mandibles from 40 female Wistar rats were used, divided into rats with ovariectomy surgery or surgical simulation. Half of the rats had the right upper incisor extracted and a soft diet was introduced for half of the animals for 30 days. After euthanasia, microtomography of the mandibles was performed for bone segmentation to construct three-dimensional models. Each mandible was subjected to a three-point bending test. The simulation by finite element method was configured according to the protocol for positioning the part on the support and force action by the load cell defined in the mechanical tests. Stress dissipation was described qualitatively on a color scale distributed in ranges of stress values. All models showed a higher concentration of stresses in the regions of force action and in the support regions, with differences in stress values and locations. Diet and dental condition interfered in the distribution of stresses, with the lateral surface of the mandible being more influenced by diet and the medial surface of the mandible by diet and dental condition.

## 1. Introduction

Osteoporosis is a systemic disease characterized by decreased mineral density and deterioration of bone microarchitecture and, consequently, patients affected by this disease are subject to fragility and fracture of this tissue [1]. Menopause is the main risk factor for osteoporosis in women, due to the decline in estrogen production, an important hormone for maintaining bone homeostasis, resulting in accelerated reabsorption due to increased osteoclast activity and deficient formation due to decreased osteoblastic activity [2].

Tooth extraction is one of the most common procedures on alveolar bones, and tooth loss is one of the most critical indicators of oral health [3]. Although rates have fallen in recent years, 158 million people are still affected by severe tooth loss [4]. In this context, the relationship between osteoporosis and tooth extraction should be further verified. Some preclinical studies have already addressed conclusions on this subject, indicating that an osteoporotic phenotype delays alveolar bone repair after tooth extraction [5,6,7].

Studies have evaluated the mandibular structure in osteoporotic patients using clinical and radiographic parameters, resulting in a decrease in jaw bone mass and density and a higher incidence of tooth loss [8,9,10].

Animal models have been remarkably useful for studies of osteoporosis. Among them, ovariectomy (OVX) has been the most common method to induce osteoporosis and is recommended by the United States Food and Drug Administration (FDA) [11].

According to Kalu [12], OVX induces a decrease in ovarian hormones in rats and an increase in intestinal calcium absorption which, as a result, generates accelerated bone loss, the same as what happens in certain postmenopausal women, due to osteoporosis. The study by Irie et al. [13] analyzed the mandibles of SHAM rats and ovariectomized rats using computed tomography and concluded that there was a loss of mandibular bone mass and trabeculation, as well as in the interradicular septum of the first molar of ovariectomized rats.

Mavropoulos et al. [14] showed that the mandibular alveolar bone is less sensitive than the proximal tibia to osteoporosis induced by estrogen deficiency in rats [14]. Studies have demonstrated a negative effect of OVX on the mandible, both when evaluating the body of the mandible [15], the condyle of the mandible [16,17], and the alveolar process [18,19]. However, there are studies that concluded that the effect of OVX on the mandible, whether on the condyle or on the alveolar bone, was significantly milder compared to other long bones [20,21,22,23,24]. These discrepancies between study results can be explained by diversity in the measurement site and rat species [25].

Masticatory mechanical load is directly related to the mandibular bony structure [26]. The exclusive consumption of a soft diet leads to masticatory hypofunction with a consequent reduction in bone mineral density. On the other hand, the consumption of a hard diet, because it requires greater chewing force, results in greater apposition of this tissue, causing the bone microarchitecture to be preserved [27].

It is known that the hardness of food is felt during chewing and affects chewing force [28] and muscle activity used in the jaw [29]. Bite force regulation occurs through mechanoreceptors that perform central control mechanisms and sensory feedback according to the appearance (size and texture) of the food ingested [30,31,32]. This control allows for an efficient reduction of food prior to swallowing [33,34].

The alveolar bone, the bony process of the jaw that provides accommodation for the dentition, is subjected to heavy forces during chewing. Kiliaridis [35], using growing rats on a calcium-deficient diet, concluded that normal masticatory function (hard diet) led to a higher rate of bone apposition and a better organized trabecular system compared to animals fed a soft diet. The correlation between mandibular alveolar trabecular architecture, bone mineral density, and chewing mechanical load was established in the study by Mavropoulos et al. [26], where chewing forces were modified by altering food consistency in adult rats. It has been suggested that reducing the chewing load of alveolar bone (soft food) leads to reduced bone mineral density, volume, and thickness of trabecular bone, as well as narrowing of the alveolar process itself. It appears that the mechanical load during chewing directly influences the macro and microarchitecture of the alveolar bone.

The finite element method (FEM) is a mathematical evaluation that aims to use computational models to determine, through simulations, displacements, stresses, and deformations. It is a satisfactory method for biomechanically evaluating bone structures to understand craniofacial biomechanics [36].

From a mechanical point of view, the loads produced by systems in bony tissue, for example, chewing, at a macroscopic level, produce tissue deformations which generate complex changes in the extracellular matrix, which in turn stimulate bone cells. In relation to the alveolar bone, when we think about chewing, studies using the finite element method [37,38,39,40,41] showed that, whether in rodents, non-human primates or humans, there is a bone adaptation resulting from different deformation regimes associated with chewing (number of chewing cycles when biting the food). The morphology, mechanical properties, positioning configuration of the three-dimensional model, load application conditions and mechanical properties are important factors in interpreting the results generated in finite element analysis (FEA) [41,42]. Recently, Ferreira et al. [40] verified the relationship between mechanical deformation patterns and the expression of Wnt/β-Catenin by immunohistochemical analysis. The study revealed that distributions of mechanical deformations by finite element analysis occur uniformly in the alveolar bone after tooth extraction, in addition to demonstrating the expression of Wnt/β-Catenin as proof that there was a bone microenvironment altered by mechanical stimuli. This study made it possible to realize that more studies are needed with in vivo and in vitro data to understand rat chewing in these experimental conditions to be applied in computational simulation using the FEA.

Mavropoulos et al. [27] demonstrated that the fact that the mandible is less sensitive to osteoporosis may be due to the mechanical load of the mandibular bone during chewing, which demonstrated an impact on mandibular bone density and microarchitecture in growth [27] and in the rat adult [26]. To test this hypothesis, the authors compared the effect of estrogen depletion (ovariectomy) and reduced chewing load (soft diet), as well as their interaction on the mandibular alveolar bone and proximal cancellous tibia in adult rats due to the anabolic effect of bone tension by chewing. Based on the hypothesis that the diet (food consistency), systemic condition such as osteoporosis and dentition may mechanically affect the mandibular bone structure, the aim of this study was to evaluate the mechanical effect of ovariectomy, diet, and tooth extraction on the alveolar bone structure of the mandible of Wistar rats.

## 2. Materials and Methods

### 2.1. Experimental Design

Forty female rats (Rattus norvegicus albinus), Wistar lineage, 4 months old (250–300 g) were used, coming from the Multidisciplinary Center for Biological Research in Science in Laboratory Animals-CEMIB-UNICAMP. They were kept in the animal research house (four animals/box) of the Piracicaba Dental School (FOP-UNICAMP), with a temperature of 22 ± 2 °C, a controlled light cycle (12 h/12 h) and free access to water and food. At a certain stage, the feed was controlled and explained below.

The rats were randomly distributed into different groups for the experiments. 20 female rats formed the Sham-operated control (SHAM) groups. The sham-operated control groups were the rats that were submitted to surgical exposure of the uterine horns and ovaries was performed without removing the ovaries.

Of these 20 rats, 5 rats had their teeth and diet maintained normal (hard diet). Five rats had their teeth kept normal and were introduced to a soft diet. Five rats underwent extraction of the right upper incisor, and the diet was maintained as normal (hard diet). Five rats underwent extraction of the right upper incisor and were introduced to a soft diet. Extractions were performed 30 days after the day of the simulated ovariectomy (OVX) surgical procedure. The introduction of the soft diet took place from the day of extraction.

Twenty rats formed the OVX groups. Of these, 5 rats had their teeth and diet maintained normal (hard diet). Five rats had their teeth kept normal and were introduced to a soft diet. Five rats underwent extraction of the right upper incisor, and the diet was maintained as normal (hard diet). Five rats underwent extraction of the right upper incisor and were introduced to a soft diet. Extractions were performed 30 days after the day of the ovariectomy (OVX) surgical procedure. The introduction of the soft diet took place from the day of extraction. Table 1 shows the distribution of groups and the euthanasia period.

### 2.2. Ovariectomy Surgery

A vaginal smear was taken from all animals to check the estrous cycle, at around 9 am, according to the Long and Evans technique [43] and analyzed fresh under an optical microscope for 15 consecutive days. After verifying that the rats were in metestrus phasis of the cycle, the rats in the OVX groups underwent ovariectomy surgery. After sedation via an intraperitoneal injection of ketamine (ketamine hydrochloride, injectable, Syntec do Brasil Ltd., Santana do Parnaíba, SP, Brazil) and xylazine (Xilazine-Coopers, Brazil, Ltd., Osasco, SP, Brazil), the rats remained immobile and were placed in lateral decubitus to make a 1 cm incision in the lateral plane on a surgical board. After the integumentary tissue, the subcutaneous tissue and the peritoneum were sectioned so that the abnominal cavity could be accessed. The ovaries and uterine horns were then located, which were exposed and lacquered with Polyglactin 910 4.0 wire (Vicryl 4.0, Johnson & Johnson, New Brunswick, NJ, USA). Then, the ovaries were removed, and the sectioned layers were sutured one by one to complete the synthesis process with Polyglactin 910 4.0 thread (Vicryl 4.0, Johnson & Johnson, New Brunswick, NJ, USA). This process was carried out on both sides (left and right) of the animal.

The rats in the SHAM groups underwent the same surgical procedure, but only surgical exposure of the uterine horns and ovaries was performed without removing the ovaries.

### 2.3. Tooth Extraction

After the OVX and SHAM procedures, the absence of the estrous cycle was verified for the rats that underwent ovariectomy and then, 30 days after ovariectomy, the rats underwent extraction of the right upper incisor.

The right upper incisor was performed under sedation via an intraperitoneal injection of (ketamine hydrochloride, injectable, Syntec do Brasil Ltd., Santana do Parnaíba, SP, Brazil) and xylazine (Xilazine-Coopers, Brazil, Ltd., Osasco, SP, Brazil). The maxilla of the rats was wiped with antiseptic (polyvinylpyrrolidone iodine, PVPI 10% Topic—Riodeine^®^, Riodeine Chemical Industry, Ltd., São José do Rio Preto, SP, Brazil) before extraction of the upper right incisor. The extraction was performed with the adapted instruments. The gingival fibromucous was then sutured with 4/0 polyglactin 910 (Vicryl), and an injection of ketoprofen (NSAID—5 mg/kg) via subcutaneous, one time, per 1 day was administered.

### 2.4. Introduction of the Soft Diet

After the right upper incisor extraction surgeries, the rats belonging to the hard diet groups continued to receive the special Purina rat food (Société des Produits Nestlé S.A.^®^, Vevey, Switzerland). The rats in the soft diet groups received a mixture of water and rat food (ground pellets) creating a soft paste [27]. The day zero on which the rats received the soft diet was counted from tooth extraction over a period of 30 days.

### 2.5. Euthanasia

After 30 days post tooth extraction, all rats were euthanized. At the time of euthanasia, a lateral incision was made in the abdomen and the horns of the uterus were analyzed in relation to appearance and atrophy. The euthanasia of the animals was carried out by overdose of the combination of anesthetics (225–300 mg/kg + 30% Ketamine Hydrochloride + Xylazine) intraperitoneally. The head of each animal was disarticulated from the body and dissected.

### 2.6. Sample Dissections

After euthanasia of the rats from all groups, the heads were dissected to obtain the mandibles of each animal. The jaws obtained were frozen because, according to Schwartz-Dabney and Dechow [44], the freezing process does not affect the elastic properties of the bone. For transport until the time of data collection on the microtomography, the bone samples were stored in a solution that maintained the physical properties of the bone, consisting of equal parts of ethanol (95%) and isotonic saline solution [45,46].

### 2.7. Sample Scanning by Microcomputed Tomography

Microtomography scan was performed on the mandible of one animal from each group, which were randomly selected, for subsequent construction of the three-dimensional geometry and finite element model for computational simulation. Thus, the mandibles were subjected to microcomputed tomography on a Skyscan 1174 Microtomography (Bruker, MA, USA), with voltage equal to 50 kV and amperage equal to 800 µA. The microtomography machine belongs to the Microscopy and Imaging Center of the Piracicaba Dental School (FOP/UNICAMP). The scan was configured to produce high-resolution images, with pixel dimensions equal to 10.2 μm and isotropic voxels. This configuration corresponds to the field of view (FOV—Field of View) adapted to the size of the mandible, as obtained from previous research carried out by the research group, to obtain the complete mandibular structure with maximum details of the bone architecture. Therefore, 8 scans were performed, obtaining 8 microcomputed tomography scans (one per group).

### 2.8. Mechanical Tests

**Figure 1:**
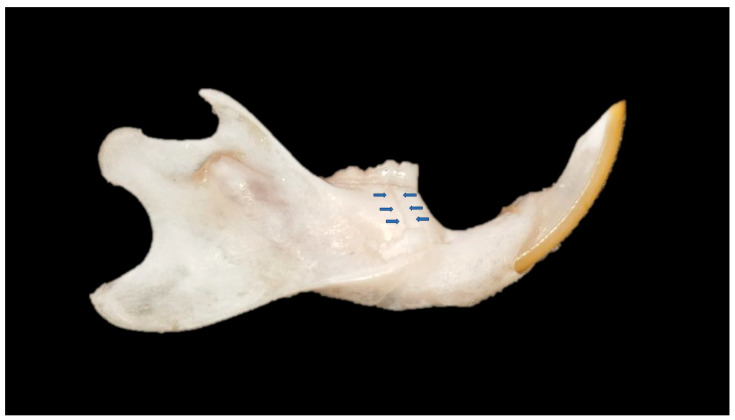
Lateral view of a rat mandible specimen after mechanical test. The arrows showed the fracture line.

All mandibles were subjected to mechanical flexion tests at 3 points using the Instron 4411 universal test equipment (Instron Corp. Norwood, MA) belonging to the Dental Materials Laboratory of the Piracicaba Dental School (FOP/UNICAMP). The mandibles were positioned horizontally with the side facing the equipment’s load cell. The load cell was activated perpendicular to the alveolar bone of each mandible in the region of the lower 1st molar. The force (N/s) collected was that at the moment of fracture (Figure 1).

### 2.9. Finite Element Analysis

#### 2.9.1. Construction of Three-Dimensional Geometry and Finite Element Model

The microtomographic images were imported into the Materialise Mimics Research v. software 18 (Materialise, Leuven, Belgium). The segmentation of the bone structure and dental structures was carried out, based on the automatic demarcation of the pixels included in the different ranges of gray scale (GV) values.

**Figure 2:**
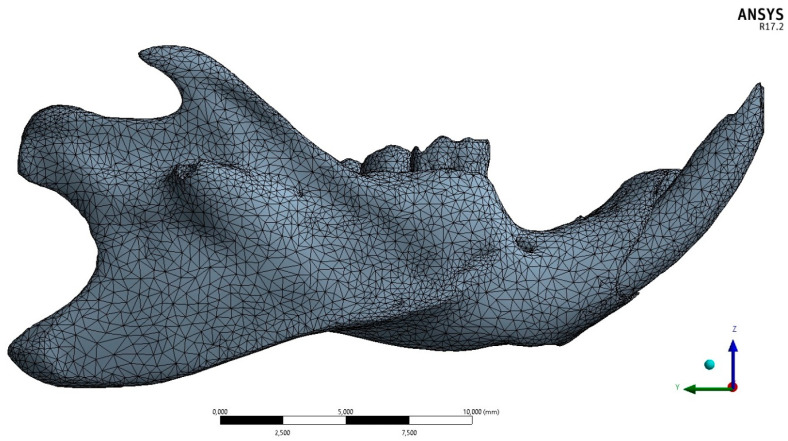
Lateral view of the finite element model showing tetrahedral elements in Ansys v17.2 software (Ansys Inc., Canonsburg, PA, USA).

After segmentation, the demarcated structures were converted into 3D polygonal surfaces (stereolithographic triangle mesh) and then transferred to the Materialise 3-Matic v software. 10 (Materialise, Leuven, Belgium) for optimization of 3D surfaces (correction of geometric errors) and conversion to volumetric meshes formed by tetrahedra, thus characterizing the finite element models (Figure 2). The dimensions of the tetrahedral elements were configured to better adapt to the models, to maintain anatomical characteristics and a geometric quality greater than 0.5 (considering a range from 0 to 1). After conversion to finite element meshes, the meshes presented an average element quality value q = 0.6.

#### 2.9.2. Computational Simulation by Finite Element Analysis

The FEA was performed to simulate the mechanical test. The finite element models were imported into the software Ansys v 17.2 (Ansys Inc., Canonsburg, EUA) and the mechanical properties of bone were assigned according the conversion of apparent density (*ρ*) to elastic modulus (E). The bone apparent density was obtained using the software Materialise Mimics research v. 18 (Materialise, Leuven, Belgium) through the segmentation on the microCT images, in which the average gray values for the pixels were considered.

To obtain an estimate of elastic modulus from apparent density, an expression obtained from flexure mechanical test [47] were used (Theorem 1), as follows:

*EC* = 2314 × *ρ*^1.57^ (1)

where *EC* is the bone elastic modulus and *ρ* is the apparent bone density. The values of *EC* were calculated for each group.

**Figure 3:**
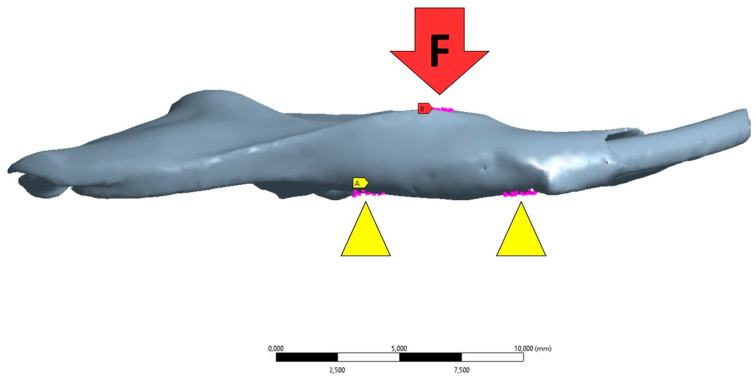
Position of finite element model following the place of support machine (yellow triangle) where the nodal displacement was restrained in all axes and force application (F) on the region corresponding to the alveolar bone of lower 1st molar.

The simulation was set up according to the mechanical test protocol of mandible positioning on the support and the force action from the load cell. These parameters were used to configure the boundary and load conditions in FEA. The boundary condition was defined through displacement restraints in all axes at the places where the machine support was applied (Figure 3). The load condition was defined on the nodes according to the place of action by the machine load cell (Figure 3). The force magnitude was applied according to the median values (Table 2) obtained from the bone fracture in the mechanical test (three-point flexure test) for each group.

**Figure 4:**
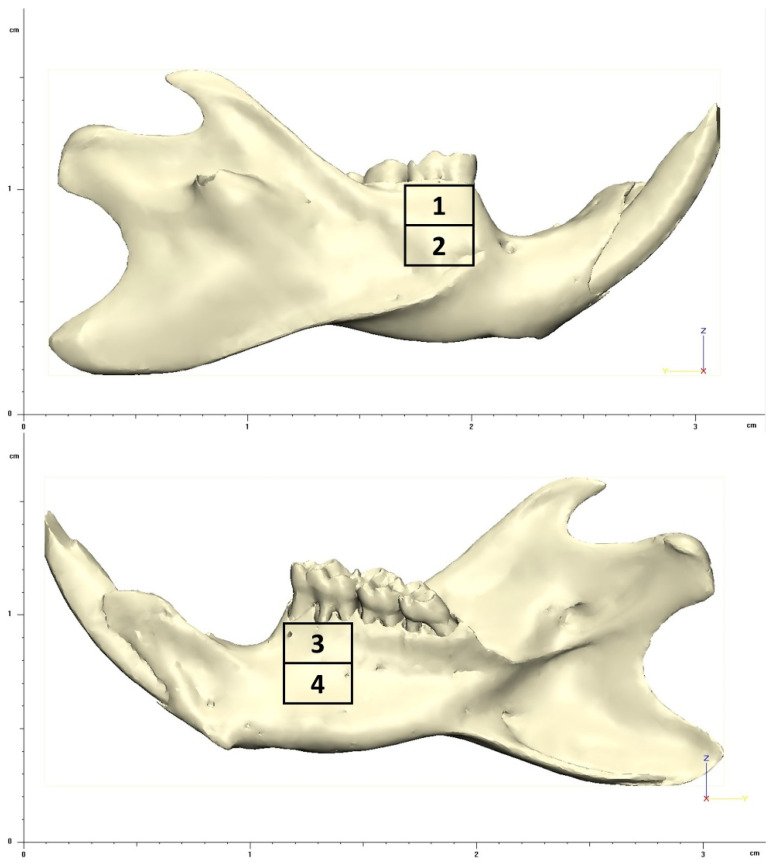
Lateral view (superior image) e medial view (inferior image) of 3D geometry of rat mandible presenting the regions considered for evaluation in FEA. The numbers 1, 2, 3, and 4 indicate the regions 1, 2, 3, and 4, respectively.

The result analysis in the post-processing was performed to calculate the equivalent von-Mises stress, with the energy dissipation from a mechanical force indicating the areas of stress concentration. The stress dissipation was described qualitatively according to a color scale, whose colors were distributed in interval of stress values in megapascal (MPa) and standardized in 4 regions as shown in Figure 4, as follows:

Region 1: buccal surface of alveolar process at lower 1st molar.

Region 2: lateral surface on the middle third at lower 1st molar.

Region 3: lingual surface of alveolar process at lower 1st molar.

Region 4: medial surface on the middle third at lower 1st molar.

## 3. Results

**Figure 5:**
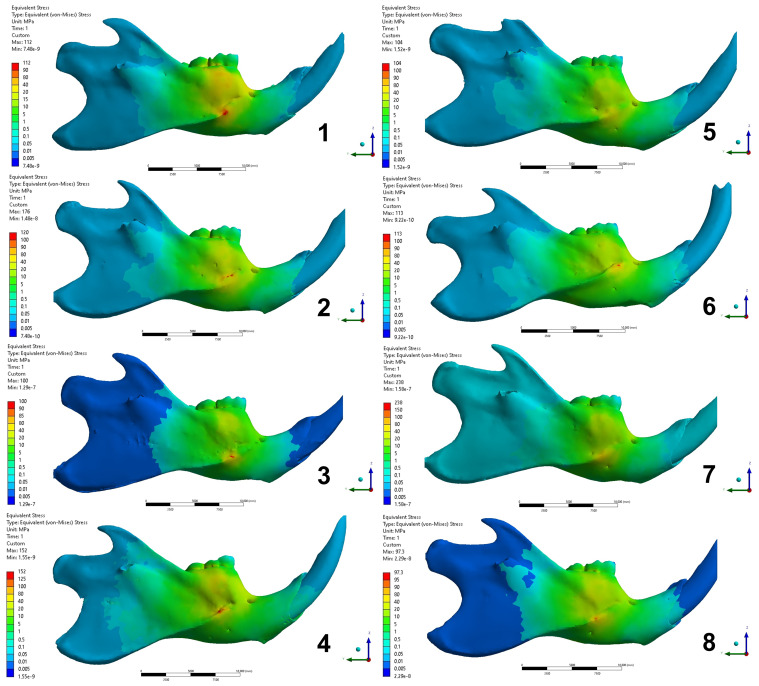
Distribution of von-Mises stress on the lateral surface of the mandibles in each group (Groups 1 to 8). 1: OVX + EXTRACTION + HARD DIET; 2: OVX + EXTRACTION + SOFT DIET; 3: OVX + NORMAL + HARD DIET; 4: OVX + NORMAL + SOFT DIET; 5: SHAM + EXTRACTION + HARD DIET; 6: SHAM + EXTRACTION + SOFT DIET; 7: SHAM + NORMAL + HARD DIET; 8: SHAM + NORMAL + SOFT DIET.

**Figure 6:**
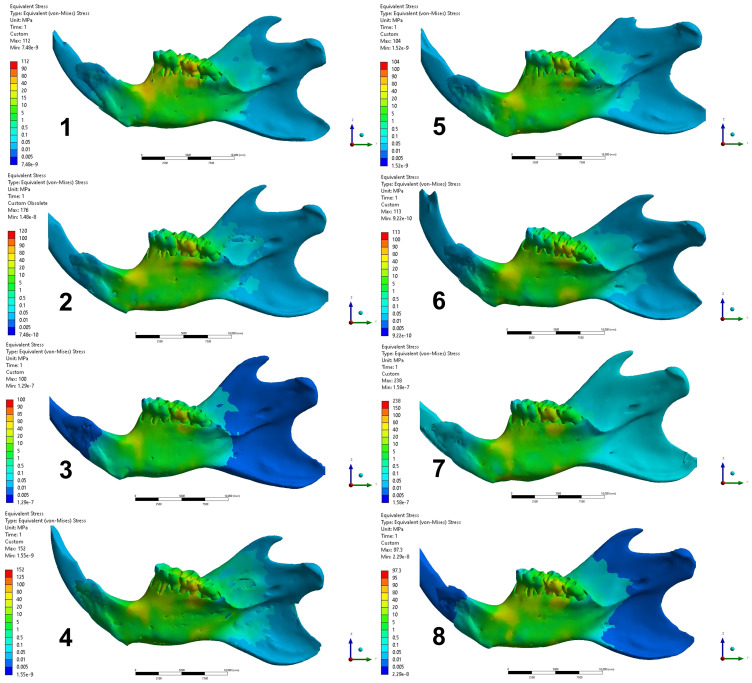
Distribution of von-Mises stress on the medial surface of the mandibles in each group (Groups 1 to 8). 1: OVX + EXTRACTION + HARD DIET; 2: OVX + EXTRACTION + SOFT DIET; 3: OVX + NORMAL + HARD DIET; 4: OVX + NORMAL + SOFT DIET; 5: SHAM + EXTRACTION + HARD DIET; 6: SHAM + EXTRACTION + SOFT DIET; 7: SHAM + NORMAL + HARD DIET; 8: SHAM + NORMAL + SOFT DIET.

The FEA simulation results were observed by qualitative analysis of the distribution of von-Mises stress in the models (Figure 5 and Figure 6). According to the configuration of the analysis, which characterized the simulation of the three-point flexural test, all models showed higher stress concentration in the regions of force action (lateral surface) and in the support regions (medial surface). The standardized regions for analysis, as described in the methods, manifested variations according to the groups.

Regions 1 and 2: group 1 showed higher stress with values ranging 40–80 MPa in region 1 and lower in region 2, ranging 15–20 MPa. Groups 2, 4, 6, and 8 showed similar stress dissipation patterns, more uniformly in regions 1 and 2. The stresses in these regions for these groups ranged 10–40 MPa. The mandible of group 3 showed the highest stress concentration in region 1, ranging 10–20 MPa, and lowest in region 2, ranging 5–10 MPa. Groups 5 and 7 showed similar patterns, with the highest stress concentration in region 2, ranged 10–20 MPa, and the lowest concentration in region 1, ranged 5–10 MPa.

Regions 3 and 4: All groups showed the same stress dissipation pattern, with a higher concentration in region 3 and lower in region 4. However, there was variation between the ranges of stress values between the groups. Groups 1, 2, 4, 5, 6 and 8 presented a stress range with values of 10–20 MPa in region 3 and stresses ranging 5–10 MPa in region 4. Groups 3 and 7 presented stresses with values ranging 5–10 MPa in region 3 and 1–5 MPa in region 4.

## 4. Discussion

The OVX rat model is the preclinical model approved by the Food and Drug Administration (FDA) [11] to study how the decline in endogenous estrogen production by the ovaries at menopause leads to postmenopausal osteoporosis and how potential interventions can preserve bone metabolism in this condition. Animals with hormone deficiency presented whitish (anemic) horns, with atrophy and thin, while those of SHAM animals were well vascularized, thick, voluminous, and pink [48]. Although the literature has demonstrated the substantial effects of estrogen deficiency on the vertebral and limb bones of the OVX rat model, its effects on the jaw have not been fully clarified [49]. It is known that chewing can play an important role in stimulating bone cell activities in various regions of the jaw [49]. The underlying mechanism of mechanobiological osteogenesis in the mandibular bone remains to be clarified. Considering these needs, the present study mechanically evaluated, through FEA, the effect of OVX on the bone structure of the mandible of Wistar rats subjected to diet change and tooth extraction.

The distribution of stresses in the bony structure in FEA may indicate greater stimulation in the bony structure, implying bone remodeling when the simulation represents physiological or pathological mechanical actions [40,41] or may indicate greater or lower mechanical resistance, when the simulation represents a biomechanical test [50,51]. In both situations, studies have shown that the results obtained in FEA establish a correlation with real conditions. Thus, from an overview of the results obtained in the computer simulation by FEA in this present study, it is understood that the analyses represented the characteristics of each group when considering the differences in relation to the type of diet (hard or soft).

From the results of the present research, the hard diet mechanically protected (adapted) the mandibular bony structure of the rats, including those with OVX induced by estrogen deficiency, as the FEA that simulated the mechanical test revealed a difference between the groups in relation to diet (Figure 5 and Figure 6). The groups of rats fed a hard diet showed different stress distribution patterns in regions 1 and 2 (lateral surface of the mandible) compared to the groups of rats fed a soft diet, which showed a uniform pattern in these regions (Figure 5). This characteristic is not observed in the distribution of stresses in regions 3 and 4 (medial surface of the mandible) (Figure 6), which can be explained by the difference in the chewing muscle insertions, since the insertions have a greater quantity of fibers in the lateral region of the mandible [52]. According to Liu et al. [53], changes in the mineral structure of the mandible due to the difference in occlusal stimulation occur mainly at the sites of muscle contraction, at the sites of reaction force and insertion of the muscles that participate in the masticatory movement. Zelig et al. [54] stated that chewing capacity is influenced by occlusion, which is related to the number and arrangement of teeth in the dental arch.

The alveolar bone is the bony process of the mandible that provides accommodation for the dentition and is subject to strong forces during chewing. The correlation between mandibular alveolar trabecular architecture, bone mineral density and masticatory mechanical load was established in a previous study, where masticatory forces were modified by altering food consistency in the adult rat [26]. It has been suggested that a reduction in the chewing load of the alveolar bone (soft food) leads to a reduction in bone mineral density, volume, and thickness of trabecular bone, as well as narrowing of the alveolar process itself. It is believed that the mechanical load during chewing directly influences the alveolar bone macro and microarchitecture.

Teeth form the occlusal area where food particles are physically broken into smaller particles to facilitate swallowing and digestion [55]. The number of processing cycles increases as food becomes more difficult to chew. Adding liquids to a dry solid food has been shown to reduce muscle activity and the number of chewing cycles until swallowing [56]. This unique biomechanical configuration is totally different from any other region of the body’s skeleton. In this context, it is understood that the condition of tooth extraction and/or soft diet, in this present study, influenced the results obtained by FEA, since the groups with tooth extraction presented a similar distribution configuration of stress in regions 3 and 4 (Figure 6). And the presence of all teeth and the hard diet reduced the concentration of stresses distributed to the mandibular bony structure. This result occurred independently of the systemic condition, in this case OVX, reinforcing the importance of maintaining masticatory function in the mechanical characteristics of the mandibular bony structure.

## 5. Conclusions

The association of the mechanical test and simulation by finite element analysis showed that the diet and dental condition interfered in the distribution of stresses on the rat mandible. The ovariectomy not influenced mechanically the alveolar mandibular bone structure. The presence of teeth shows a protective mechanical effect on the mandible.

## Figures and Tables

**Table 1:** Description of groups. OVX: ovariectomy. NORMAL: Without tooth extraction.

Group	Rats per Group	Description
1	5	**OVX + EXTRACTION + HARD DIET**
2	5	**OVX + EXTRACTION + SOFT DIET**
3	5	**OVX + NORMAL + HARD DIET**
4	5	**OVX + NORMAL + SOFT DIET**
5	5	**SHAM + EXTRACTION + HARD DIET**
6	5	**SHAM + EXTRACTION + SOFT DIET**
7	5	**SHAM + NORMAL + HARD DIET**
8	5	**SHAM + NORMAL + SOFT DIET**

**Table 2:** Median from force magnitude (N) obtained in each group at the fracture moment from three-point flexure test.

GROUPS								
	1	2	3	4	5	6	7	8
Median	136.6	126.0	104.0	124.2	103.6	93.74	141.3	152.4

## Data Availability

The datasets used and analyzed in the current study are available from the corresponding author upon reasonable request.

## References

[1] Pavone V., Testa G., Giardina S.M.C., Vescio A., Restivo D.A., Sessa G. (2017). Pharmacological Therapy of Osteoporosis: A Systematic Current Review of Literature. Front. Pharmacol..

[2] Esteall R., O’Neill T.W., Hofbauer L.C., Langdahl B., Reid I.R., Gold D.T., Cummings S.R. (2016). Postmenopausal osteoporosis. Nat Rev. Dis. Primers..

[3] Haworth S., Shungin D., Kwak S.Y., Kim H.Y., West N.X., Thomas S.J., Franks P.W., Timpson N.J., Shin M.-J., Johansson I. (2018). Tooth loss is a complex measure of oral disease: Determinants and methodological considerations. Community Dent. Oral Epidemiol..

[4] Kassebaum N.J., Bernabé E., Dahiya M., Bhandari B., Murray C.J.L., Marcenes W. (2014). Global burden of severe tooth loss: A systematic review and meta-analysis. J. Dent. Res..

[5] Arioka M., Zhang X., Li Z., Tulu U.S., Liu Y., Wang L., Yuan X., Helms J.A. (2019). Osteoporotic changes in the periodontium impair alveolar bone healing. J. Dent. Res..

[6] de Oliveira Puttini I., Gomes-Ferreira P.H.D.S., de Oliveira D., Hassumi J.S., Gonçalves P.Z., Okamoto R. (2019). Teriparatide improves alveolar bone modelling after tooth extraction in orchiectomized rats. Arch. Oral Biol..

[7] Miranda T.S., Napimoga M.H., De Franco L., Marins L.M., de Malta F.S., Pontes L.A., Morelli F.M., Duarte P.M. (2020). Strontium ranelate improves alveolar bone healing in estrogen-deficient rats. J. Periodontol..

[8] Alam T., AlShahrani I., Assiri K.I., Almoammar S., Togoo R.A., Luqman M. (2020). Evaluation of clinical and radiographic parameters as dental indicators for postmenopausal osteoporosis. Oral Health Prev. Dent..

[9] Kribbs P.J., Chesnut C.H., Ott S.M., Kilcoyne R.F. (1989). Relationships between mandibular and skeletal bone in an osteoporotic population. J. Prosthet. Dent..

[10] Kribbs P.J. (1990). Comparison of mandibular bone in normal and osteoporotic women. J. Prosthet. Det..

[11] Thompson D.D., Simmons H.A., Pirie C.M., Ke H.Z. (1995). FDA Guidelines and animal models for osteoporosis. Bone.

[12] Kalu D.N. (1991). The ovariectomized rat model of postmenopausal bone loss. Bone Miner..

[13] Irie K., Sakakura Y., Tsuruga E., Hosokawa Y., Yajima T. (2004). Three-dimensional Changes of the Mandible and Alveolar Bone in the Ovariectomized Rat Examined by Micro-focus Computed Tomography. J. Jpn. Soc. Periodontol..

[14] Mavropoulos A., Rizzoli R., Ammann P. (2007). Different responsiveness of alveolar and tibial bone to bone loss stimuli. J. Bone Miner..

[15] Elovic R.P., Hipp J.A., Hayes W.C. (1995). Ovariectomy decreases the bone area fraction of the rat mandible. Calcif. Tissue Int..

[16] Tanaka M., Ejiri S., Kohno S., Ozawa H. (2000). Region-specific bone mass changes in rat mandibular condyle following ovariectomy. J. Dent. Res..

[17] Sakakura Y., Shide N., Tsuruga E., Irie K., Yajima T. (2001). Effects of running exercise on the mandible and tibia of ovariectomized rats. J. Bone Miner. Metab..

[18] Hara T., Sato T., Oka M., Mori S., Shirai H. (2001). Effects of ovariectomy and/or dietary calcium deficiency on bone dynamics in the rat hard palate, mandible and proximal tibia. Arch. Oral Biol..

[19] Tanaka M., Toyooka E., Kohno S., Ozawa H., Ejiri S. (2003). Long-term changes intrabecular structure of aged rat alveolar bone after ovariectomy. Oral Surg. Oral Med. Oral. Pathol. Oral Radiol. Endod..

[20] Fujita T., Kawata T., Tokimasa C., Tanne K. (2001). Influence of oestrogen and androgen on modelling of the mandibular condylar bone in ovariectomized and orchiectomized growing mice. Arch. Oral Biol..

[21] Yamashiro T., Takano-Yamamoto T. (1998). Differential responses of mandibularcondyle and femur to oestrogen deficiency in young rats. Arch. Oral Biol..

[22] Tanaka M., Ejiri S., Nakajima M., Kohno S., Ozawa H. (1999). Changes of cancellous bone mass in rat mandibular condyle following ovariectomy. Bone.

[23] Johnson R.B., Gilbert J.A., Cooper R.C., Parsell D.E., Stewart B.A., Dai X., Nick T.G., Streckfus C.F., Butler R.A., Boring J.G. (2002). Effect of estrogen deficiency onskeletal and alveolar bone density in sheep. J. Periodontol..

[24] Jiang G., Matsumoto H., Fujii A. (2003). Mandible bone loss in osteoporosis rats. J. Bone Miner. Metab..

[25] DeMoss D.L., Wright G.L. (1998). Sex and strain differences in whole skeletal development in the rat. Calcif. Tissue Int..

[26] Mavropoulos A., Odman A., Ammann P., Kiliaridis S. (2010). Rehabilitation of masticatory function improves the alveolar bone architecture of the mandible in adult rats. Bone.

[27] Mavropoulos A., Kiliaridis S., Rizzoli R., Ammann P. (2014). Normal masticatory function partially protects the rat mandibular bone from estrogen-deficiency induced osteoporosis. J. Biomech..

[28] Kohyama K., Hatakeyama E., Sasaki T., Dan H., Azuma T., Karita K. (2004). Effects of sample hardness on human chewing force: A model study using silicone rubber. Arch. Oral Biol..

[29] Agrawal K.R., Lucas P.W., Bruce I.C., Prinz J.F. (1998). Food properties that influence neuromuscular activity during human mastication. J. Dent. Res..

[30] Woda A., Mishellany A., Peyron M.A. (2006). The regulation of masticatory functionand food bolus formation. J. Oral Rehabil..

[31] Kohyama K., Sakai T., Dan H. (2003). Active stress during compression testing ofvarious foods measured using a multiple-point sheet sensor. Biosci. Biotechnol. Biochem..

[32] Van der Bilt A. (2010). Assessment of mastication with implications for oral rehabilitation: A review. J. Oral Rehabil..

[33] Peyron M.A., Mishellany A., Woda A. (2004). Particle size distribution of foodboluses after mastication of six natural foods. J. Dent. Res..

[34] Mishellany A., Woda A., Labas R., Peyron M.A. (2006). The challenge of mastica-tion: Preparing a bolus suitable for deglutition. Dysphagia..

[35] Kiliaridis S. (1989). Muscle function as a determinant of mandibular growth innormal and hypocalcaemic rat. Eur. J. Orthod..

[36] Richmond B.G., Wright B.W., Grosse I., Dechow P.C., Ross C.F., Spencer M.A., Strait D.S. (2005). Finite element analysis in functional morphology. Anat. Rec. A Discov. Mol. Cell. Evol. Biol..

[37] Cox P.G., Rayfield E.M., Fagan M.J., Herrel A., Pataky T.C., Jeffery N. (2012). Functional evolution of the feeding system in rodents. PLoS ONE.

[38] Ross C.F., Iriarte-Diaz J., Reed D.A., Stewart T.A., Taylor A.B. (2016). In vivo bone strain in the mandibular corpus of Sapajus during a range of oral food processing behaviors. J. Hum. Evol..

[39] Prado F.B., Freire A.R., Rossi A.C., Ledogar J.A., Smith A.L., Dechow P.C., Strait D.S., Voigt T., Ross C.F. (2016). Review of In Vivo Bone Strain Studies and Finite Element Models of the Zygomatic Complex in Humans and Nonhuman Primates: Implications for Clinical Research and Practice. Anat. Rec..

[40] Ferreira B.C., Freire A.R., Araujo R., do Amaral-Silva G.K., Okamoto R., Prado F.B., Rossi A.C. (2020). β-catenin and Its Relation to Alveolar Bone Mechanical Deformation—A Study Conducted in Rats with Tooth Extraction. Front Physiol..

[41] Rossi A.C., Freire A.R., Ferreira B.C., Faverani L.P., Okamoto R., Prado F.B. (2021). Effects of premature contact in maxillary alveolar bone in rats: Relationship between experimental analyses and a micro scale FEA computational simulation study. Clin. Oral Investig..

[42] Berthaume M.A., Dechow P.C., Iriarte-Diaz J., Ross C.F., Strait D.S., Wang Q., Grosse I.R. (2012). Probabilistic finite element analysis of a craniofacial finite element model. J. Theor. Biol..

[43] Long J.A., Evans H.M.L. (1922). The Oestrous Cycle in the Rat and It’s Associated Phenomena.

[44] Schwartz-Dabney C.L., Dechow P.C. (2003). Variations in cortical material properties throughout the human dentate mandible. Am. J. Phys. Anthropol..

[45] Ashman R.B., Cowin S.C., Van Buskirk W.C., Rice J.C. (1984). A continuous wave technique for the measurement of the elastic properties of cortical bone. J. Biomech..

[46] Zioupos P., Smith C.W., An Y.H., An R.A., Draughn R.A. (2000). Factors affecting mechanical properties of bone. Mechanical Testing of Bone and the Bone-Implant Interface.

[47] Wirtz D.C., Schiffers N., Pandorf T., Radermacher K., Weichert D., Forst R. (2000). Critical Evaluation of Known Bone Material Properties to Realize Anisotropic FE-Simulation of the Proximal Femur. J. Biomech..

[48] Scalize P.H., de Sousa L.G., Regalo S.C., Semprini M., Pitol D.L., da Silva G.A., de Almeida Coelho J., Coppi A.A., Laad A.A., Prado K.F. (2015). Low-level laser therapy improves bone formation: Stereology findings for osteoporosis in rat model. Lasers Med. Sci..

[49] Inoue M., Ono T., Kameo Y., Sasaki F., Ono T., Adachi T., Nakashima T. (2019). Forceful mastication activates osteocytes and builds a stout jawbone. Sci. Rep..

[50] Iwai T., Hoshi M., Oebisu N., Orita K., Shimatani A., Takada N., Nakamura H. (2021). Prediction of Risk Factors for Pathological Fracture After Bone Tumor Biopsy Using Finite Element Analysis. Cancer Manag. Res..

[51] Hudyma N., Lisjak A., Tatone B.S., Garner H.W., Wight J., Mandavalli A.S., Olutola I.A., Pujalte G.G.A. (2022). Comparison of Cortical Bone Fracture Patterns Under Compression Loading Using Finite Element-Discrete Element Numerical Modeling Approach and Destructive Testing. Cureus.

[52] Haddad J. (2018). Morphological Characteristics of Masticatory Muscles in Wistar Rats (Rattus Norvegicus Albinus) by Computed Microtomography. Master’s Thesis.

[53] Liu J., Liu S.Y., Zhao Y.J., Gu X., Li Q., Jin Z.L., Chen Y.J. (2016). Effects of occlusion on mandibular morphology and architecture in rats. J. Surg. Res..

[54] Zelig R., Jones V.M., Touger-Decker R., Hoskin E.R., Singer S.R., Byham-Gray L., Radler D.R., Rothpletz-Puglia P. (2019). The Eating Experience: Adaptive and Maladaptive Strategies of Older Adults with Tooth Loss. JDR Clin. Trans. Res..

[55] van der Bilt A., Engelen L., Pereira L.J., van der Glas H.W., Abbink J.H. (2006). Oral physiology and mastication. Physiol. Behav..

[56] van der Bilt A., Engelen L., Abbink J., Pereira L.J. (2007). Effects of adding fluids to solid foods on muscle activity and number of chewing cycles. Eur. J. Oral Sci..

